# Proteomics unite traditional toxicological assessment methods to evaluate the toxicity of iron oxide nanoparticles

**DOI:** 10.3389/fphar.2022.1011065

**Published:** 2022-09-12

**Authors:** Junyuan Han, Yongzhang Tian, Minghan Wang, Yajuan Li, Jiye Yin, Wensheng Qu, Changhui Yan, Rigao Ding, Yongbiao Guan, Quanjun Wang

**Affiliations:** State Key Laboratory of Toxicology and Medical Countermeasures, Institute of Pharmacology and Toxicology, Beijing, China

**Keywords:** iron oxide nanoparticles, toxicity, lysosome, proteomics, autophagy, AKT/mTOR/TFEB signaling pathway

## Abstract

Iron oxide nanoparticles (IONPs) are the first generation of nanomaterials approved by the Food and Drug Administration for use as imaging agents and for the treatment of iron deficiency in chronic kidney disease. However, several IONPs-based imaging agents have been withdrawn because of toxic effects and the poor understanding of the underlying mechanisms. This study aimed to evaluate IONPs toxicity and to elucidate the underlying mechanism after intravenous administration in rats. Seven-week-old rats were intravenously administered IONPs at doses of 0, 10, 30, and 90 mg/kg body weight for 14 consecutive days. Toxicity and molecular perturbations were evaluated using traditional toxicological assessment methods and proteomics approaches, respectively. The administration of 90 mg/kg IONPs induced mild toxic effects, including abnormal clinical signs, lower body weight gain, changes in serum biochemical and hematological parameters, and increased organ coefficients in the spleen, liver, heart, and kidneys. Toxicokinetics, tissue distribution, histopathological, and transmission electron microscopy analyses revealed that the spleen was the primary organ for IONPs elimination from the systemic circulation and that the macrophage lysosomes were the main organelles of IONPs accumulation after intravenous administration. We identified 197 upregulated and 75 downregulated proteins in the spleen following IONPs administration by proteomics. Mechanically, the AKT/mTOR/TFEB signaling pathway facilitated autophagy and lysosomal activation in splenic macrophages. This is the first study to elucidate the mechanism of IONPs toxicity by combining proteomics with traditional methods for toxicity assessment.

## 1 Introduction

Iron oxide nanoparticles (IONPs) are used for a wide variety of biomedical and bioengineering applications, such as magnetic resonance imaging, drug delivery, cancer therapy, hyperthermia, and tissue repair, because of their unique physical and chemical properties (Anderson et al., 2019; Soetaert et al., 2020). Some of the IONPs evaluated in preclinical and clinical trials have been approved by the European Medicines Agency and United States Food and Drug Administration for the treatment of iron deficiency in chronic kidney disease and imaging of liver lesions and lymph node metastasis (Bobo et al., 2016). However, several approved IONPs-based imaging agents were later withdrawn because of severe toxic effects (Frtus et al., 2020). Emerging evidence indicates that traditional preclinical safety models might neglect secondary toxic effects, leading to inadequate comprehension of the mechanisms by which IONPs act at the cellular and subcellular levels (Frtus et al., 2020; Chrishtop et al., 2021). Although numerous *in vitro* and *in vivo* studies have explored the mechanisms of IONPs-mediated toxicities such as neurotoxicity, immunotoxicity, and cardiovascular toxicity (Mahmoudi et al., 2012; Chrishtop et al., 2021), the results have been inconsistent or even contradictory. Indeed, IONPs are known to affect multiple organelles and signaling pathways to mediate different biological responses (Zhang et al., 2016). Therefore, the mechanisms underlying IONPs-mediated toxicity should be investigated using an integrative and systematic approach.

As an emerging branch of life science research, proteomics is a high-throughput technology with high sensitivity. Several proteomics studies have revealed the toxic effects of nanomaterials and the underlying mechanisms (Frohlich, 2017; Zhang et al., 2018). New toxicological mechanisms of nanomaterials at the protein level can be revealed based on the analysis of the whole proteome using this approach (Matysiak et al., 2016). In recent years, proteomics studies have revealed several novel toxicological mechanisms of IONPs. For example, Askri et al. (2019a) used proteomics techniques to show that IONPs affected signaling pathways regulating cytoskeleton, apoptosis, and carcinogenesis in SH-SY5Y cells. However, compared to various *in vitro* proteomics studies investigating nanoparticles (Nath Roy et al., 2016), to our knowledge, only one *in vivo* proteomics study examined the effects of IONPs in the liver, brain, and lungs after intranasal exposure (Askri et al., 2019b). In fact, proteomics techniques should be considered a powerful tool to identify potential biomarkers and to evaluate toxicity following IONPs exposure. Furthermore, the identification of differentially expressed proteins (DEPs) after IONPs exposure can aid in gaining a deep insight into the mechanisms of toxicity. However, no study to date has examined the changes in the proteome following the administration of IONPs through clinically used routes, especially intravenous injection. Furthermore, no study has investigated the effects of IONPs using proteomics in combination with traditional preclinical safety assessment methods.

In the present study, we aimed to evaluate IONPs toxicity following intravenous administration for 14 days in rats using emerging proteomics methods in combination with traditional toxicological assessment methods, including hematology, serum biochemistry, histopathology, and toxicokinetics. Furthermore, we aimed to reveal the molecular mechanisms of IONPs toxicity by comparing traditional toxicological and proteomics parameters in this animal model.

## 2 Material and methods

### 2.1 Physicochemical characteristics of injected iron oxide nanoparticles

Injected IONPs in the form of polyethylene glycol-coated Fe_3_O_4_ nanoparticles in water, which contained a concentration of Fe 10 mg/ml, were kindly provided by Mingyuan Gao and Jianfeng Zeng from Soochow University, China. The IONPs were prepared using the flow synthesis method as previously reported (Jiao et al., 2015). The characteristic morphology of injected IONPs was determined using transmission electron microscopy (TEM, Tecnai G20, FEI, United States) operating at an acceleration voltage of 200 kV. The zeta potential and hydrodynamic particle size of injected IONPs were measured at 25°C using dynamic light scattering with a particle size analyzer (Zetasizer Nano ZS90, Malvern Instruments, United Kingdom).

### 2.2 Animal care

In total, 62 male and 62 female specific pathogen-free Sprague-Dawley rats aged 7 weeks were purchased from Vital River Laboratory Animal Technology (Beijing, China). The animals were dosed after one week of acclimation. During the study, all procedures for the care and use of animals were reviewed and approved by the Institutional Animal Care and Use Committee of National Beijing Center for Drug Safety Evaluation and Research (IACUC-2021-009). The laboratory animal program is fully accredited by the Association for Assessment and Accreditation of Laboratory Animal Care.

### 2.3 Study design

IONPs were intravenously administrated through the tail vein. IONPs were diluted to desired concentrations in 5% glucose solution (dissolved by sterile water) under sterile conditions. Animals were randomly assigned to four groups with 11 rats per sex (rats/sex) for vehicle group and 17 rats/sex which six rats/sex served as toxicokinetic animals in IONPs treated groups. IONPs at doses of 10, 30, and 90 mg/kg or the vehicle (5% glucose solution) were administered once daily for 14 days. The parameters evaluated during the in-life phase included clinical observations such as mortality, moribundity, general health, and signs of toxicity (once daily); food consumption (twice weekly); and body weight (twice weekly).

### 2.4 Clinical chemistry, hematology, and coagulation

Clinicopathological evaluations, including clinical chemistry, hematology, and coagulation parameters, were conducted on 5 rats per sex per group after intravenous IONPs administration for 14 days. In all animals, blood samples were collected from the inferior vena cava with the animal anesthetized using pentobarbital at the time of sacrifice. Serum chemistry parameters were analyzed using an auto analyzer (Hitachi 7,180, Hitachi). Hematological parameters were analyzed using an automatic hematology analyzer (Sysmex XN-1000v, Sysmex). Coagulation parameters were analyzed using an automated coagulation analyzer (Sysmex CS-5100, Sysmex).

### 2.5 Determination of organ coefficients

In all animals, body weight was measured immediately before sacrifice. A full necropsy was conducted in all animals, and the brain, heart, liver, spleen, and kidney were removed and immediately weighed in 5 rats/sex/group. Organ coefficients were calculated as the ratio of tissue wet weight (mg) to body weight (g).

### 2.6 Histopathological examination

The following organs were collected from 5 rats/sex/group and fixed in 10% neutral-buffered saline: brain, heart, liver, spleen, kidneys, lungs, thymus, adrenal glands, trachea, esophagus, aorta, thyroid, and parathyroid glands, pancreas, stomach, small and large intestines, mesenteric lymph nodes, uterus, vagina, ovaries, prostate gland, epididymis, seminal vesicles, urinary bladder, biceps femoris muscle, femur and sternum with bone marrow, sciatic nerve, mammary glands, and skin. Eyes with Harderian glands and testes were fixed in modified Davidson’s fixative and transferred to 10% neutral-buffered formalin within 24–48 h. Paraffin-embedded sections (3–4 μm) were prepared from all tissues and stained with hematoxylin and eosin. Microscopic examination was conducted for all tissues in all groups. Additionally, spleen sections were stained with Perls’ Prussian blue for iron detection.

### 2.7 Immunohistochemistry and immunofluorescence

For immunofluorescent staining for light chain 3 (LC3) in spleen sections, nonspecific binding was blocked with 5% goat serum (cat. No: SL038, Solarbio Life Sciences), followed by immunolabeling using a primary antibody against LC3 (cat. No: 4,108; Cell Signaling Technology) and a secondary antibody conjugated to fluorescein isothiocyanate (cat. No: ZF-0311, ZSGB-BIO). Immunofluorescence images were acquired using a 3DHISTECH P250 FLASH confocal microscope with a ×63 objective. For immunohistochemistry, tissue sections were incubated with a primary antibody against lysosome-associated membrane protein (LAMP) 2 (cat. No: 125,068, Abcam) or ferritin heavy chain 1 (FTH1, cat. No: 4,393, Cell Signaling Technology) and visualized using a streptavidin–biotin immunoenzymatic antigen detection system (cat. No: PV-9000, ZSGB-BIO).

### 2.8 TEM and TEM-energy-dispersive spectrometer analysis

Spleens collected at sacrifice were cut into approximately 1-mm3 pieces, fixed in 2.5% glutaraldehyde in 0.1 M phosphate buffer, and postfixed with 1% osmium tetroxide. After washing in double distilled H2O, the samples were gradually dehydrated in ethanol, embedded in epoxy resin, and sliced into 70-nm-thick sections. The sections were observed under bright field using an electron microscope (JEM-7610EX, JEOL, Japan) operated at 80 kV.

The samples prepared from the animals in the vehicle and 90 mg/kg IONPs groups were also analyzed to determine the element distribution of iron using TEM (JEM-2100, JEOL, Japan) equipped with an energy-dispersive spectrometry (EDS) device. The TEM images were captured at 200 kV and used for qualitative elemental analysis of the energy-dispersive spectra of the whole area.

### 2.9 Toxicokinetic and tissue distribution analyses

In animals used for toxicokinetic analysis (three rats per sex in IONPs treated groups), approximately 0.25 ml of whole blood was collected via jugular vein puncture prior to; 5, 15, and 30 min; and 1, 3, 6, and 24 h after the first (day 1) and last (day 14) doses. The collected blood samples were maintained at room temperature (18–26°C) for 30–60 min, followed by centrifugation at 1800 *g* for 10 min at 4°C to obtain serum samples, which were stored at −80°C. Following sacrifice, the spleen, liver, lungs, mesenteric lymph nodes, kidneys, heart, and brain (cerebellum and cerebrum) from three rats/sex/group were dissected and stored at −80°C. Concentrations of IONPs (expressed as total iron, ng/mL) of three rats/sex/group were determined using an inductively coupled plasma (ICP)-mass spectrometry (MS) method. The lower limits of quantitation were 25 and 75 ng/ml for serum and tissue samples, respectively. The WinNonlin software package was used for the calculation of serum toxicokinetic parameters, and the predose value was subtracted prior to the calculation.

### 2.10 Tandem mass tag-labeled quantitative proteomics

Proteomics analysis of the spleen tissue samples of the vehicle and 90 mg/kg IONPs groups was performed using the tandem mass tag (TMT)-labeled quantitative proteomic approach. The procedures included protein sample preparation, proteolysis and TMT labeling, liquid chromatography-tandem MS and high-performance liquid chromatograph analysis, and proteomics data analysis. Briefly, the spleen tissues of 12 animals (6 animals/group) were collected and ground into powder with liquid nitrogen. Lysis buffer supplemented with 1 mM phenylmethylsulfonyl fluoride (cat. No: A610425-0005, Sangon Biotech) was added to the samples, which were sonicated on ice for 3 min, followed by centrifugation twice at 12,000 *g* for 10 min at room temperature to collect the supernatants. The protein concentrations were confirmed using a bicinchoninic acid protein assay kit (cat. No: 23,225, Thermo Fisher Scientific) per the manufacturer’s descriptions. Sodium dodecyl sulfate-polyacrylamide gel electrophoresis was performed for protein quality control prior to proteomic experiments. The protein solutions were digested with trypsin, reduced/alkylated, precipitated with acetone, and enzymatically hydrolyzed at 37°C. The obtained peptides were reconstituted and labeled using a TMT kit (cat. No: A44520, Thermo Fisher Scientific). The labeled peptides were fractionated using a high-performance liquid chromatograph (Agilent 1,100 series, Aglient) with an Agilent Zorbax Extend C18 column (5 μm, 150 mm × 2.1 mm). The tryptic peptides were dissolved in mobile phase A (aqueous solution containing 0.1% formic acid) and injected into Q Exactive MS (Thermo Fisher Scientific) for separation using liquid chromatography-tandem MS. The liquid phase gradient was set as follows: 0–1 min, 2%–6% mobile phase B (acetonitrile solution containing 0.1% formic acid); 1–49 min, 6%–25% mobile phase B; 49–54 min, 25%–35% mobile phase B; 54–56 min, 35%–90% mobile phase B; and 56–60 min, 90% mobile phase B. The flow rate was maintained at 350 nL/min. Full MS scanning was acquired in a mass resolution of 120,000 with a mass range of 350–1,650 m/z, and the target automatic gain control was set at 3e6. Fifteen most intense peaks in MS were fragmented using higher energy collisional dissociation with a collision energy of 32. The MS/MS spectra were obtained with a resolution of 60,000, target automatic gain control of 1e5, and max injection time of 55 ms. The Q Exactive dynamic exclusion time was set to 40 s and run in positive mode.

### 2.11 Database search and bioinformatics analysis

Proteome Discoverer v2.4 (Thermo Fisher Scientific) was used to thoroughly search the experimental data against the UniProt database for *Rattus norvegicus* (https://www.uniprot.org, database released in July 2021, uniprot-proteome_UP000002494) with trypsin digestion specificity. The search settings were as follows: tolerance of first-stage precursor iron mass error, 10 ppm; number of missed cleavages, 2; and mass error tolerance of the secondary fragment ion, 0.02 Da. Cysteine alkylation was set as a fixed modification. False discovery rate (FDR) was adjusted to <1%, and the quantification of protein groups required ≥2 peptides. The screening criteria for DEPs with significance were a *p* value of <0.05 and a fold change of >2 (90 mg/kg IONPs group vs. vehicle group). The Gene Ontology (GO) database (http://www.geneontology.org/, released in July 2021) was used for GO analysis. The DEPs were annotated into three ontological categories (biological process, molecular function, and cellular component) using GO terms. Gene-related metabolic pathways were identified using the Kyoto Encyclopedia of Genes and Genomes (KEGG) database (http://www.genome,jp.kegg/, released in July 2021). GO and KEGG pathway enrichment analysis based on hypergeometric distribution R. For each category, a two-tailed Fisher’s exact test was used to test the enrichment of DEPs. The GO terms or the KEGG pathways with corrected *p* values of <0.05 were considered significant. An enrichment score was calculated based on the formula of $\left(\frac{m}{n}\right)/\left(\frac{M}{N}\right)$ to indicate the number of DEPs enriched to this GO term or KEGG pathway. In this formula, “N” is the number of GO/KEGG annotations in rat proteins; “n” is the number of GO/KEGG annotations in differential proteins; “M” is the number of a specific GO terms or KEGG pathways annotated in the protein of rats; “m” is the number of DEPs annotated as a specific GO term or KEGG pathway.

### 2.12 Parallel reaction monitoring for protein expression verification

The abundances of LAMP1 and Niemann–Pick C1 (NPC1), two of the DEPs identified in the 90 mg/kg IONPs group, were validated using parallel reaction monitoring of all groups administered IONPs as well as the vehicle group. The experimental and data analysis methods were previously described by Chen et al. (2021).

### 2.13 Western blot analysis

Western blotting was performed in the spleen the of 10, 30, and 90 mg/kg IONPs treated and vehicle groups. The expression levels of LAMP2, FTH1, LC3, AKT, phosphorylated (p)-AKT, mammalian target of rapamycin (mTOR), p-mTOR, transcription factor EB (TFEB), and p-TFEB were detected. The rabbit anti-LAMP2 antibody (ab125068, 1:2000) was purchased from Abcam. The rabbit antibodies against LC3 (cat. No: 4,108; 1:1,000), FTH1 (cat. No: 4,393; 1:1,000), AKT (cat. No: 4,691; 1:1,000), p-AKT (cat. No: 4,060; 1:1,000), mTOR (cat. No: 2,983; 1:1,000), p-mTOR (cat. No: 5,536; 1:1,000), TFEB (cat. No: 83,010; 1:1,000), glyceraldehyde-3-phosphate dehydrogenase (cat. No: 5,174; 1:1,000), and *β*-actin (cat. No: 8,457; 1:3,000) were purchased from Cell Signaling Technology. The rabbit polyclonal p-TFEB antibody (cat. No: PA5-114662; 1:1,000) was purchased from Thermo Fisher Scientific. Briefly, the spleens of three rats/sex/group were homogenized and lysed in radioimmunoprecipitation assay buffer containing phosphatase and protease inhibitors. The lysates were centrifugated at 12,000 *g* for 10 min at 4°C, and protein concentrations were determined using the bicinchoninic acid protein assay kit (cat. No: 23,225, Thermo Fisher Scientific). Equal amounts of protein lysates were separated on sodium dodecyl sulfate-polyacrylamide gels and transferred to polyvinylidene difluoride membranes, which were blocked with 5% nonfat dry milk in Tris-buffered saline containing 0.05% Tween 20 for 1 h at room temperature. Next, the membranes were incubated with primary antibodies in primary antibody dilution buffer (cat. No: P0023A; Beyotime) overnight at 4°C. After washing three times with Tris-buffered saline containing 0.05% Tween 20, 5 min each, the membranes were incubated with anti-rabbit immunoglobulin G antibody (cat. No: 7,074; 1:10,000; Cell Signaling Technology) for 1 h at room temperature. The signals were detected by incubating the membranes with an enhanced chemiluminescence reagent (cat. No: 34,577; Thermo Fisher Scientific), and band intensities were analyzed using ImageJ.

### 2.14 Statistical analysis

Data were expressed as means ± standard deviation and analyzed using GraphPad Prism 5.0 (GraphPad, San Diego, CA, United States). One-way analysis of variance, followed by Dunnett’s test, was used for all statistical analysis. A *p* value of <0.05 was considered to indicate statistical significance.

## 3 Results

### 3.1 Characterization of the injected iron oxide nanoparticles

The TEM images of the injected IONPs and the corresponding particle size distribution are shown in Supplementary Figures S1A,B, respectively. The average particle size was 3.6 ± 0.3 nm (Supplementary Figure S1C). The zeta potential of IONPs was −7 mV.

### 3.2 General physiological findings

The careful monitoring of the clinical signs and symptoms throughout the 14-days consecutive dosing period revealed slight piloerection and red perirhinal discharge in the 90 mg/kg IONPs group. No abnormal clinical signs were observed in the vehicle group and the 10 and 30 mg/kg IONPs groups.

**FIGURE 1:**
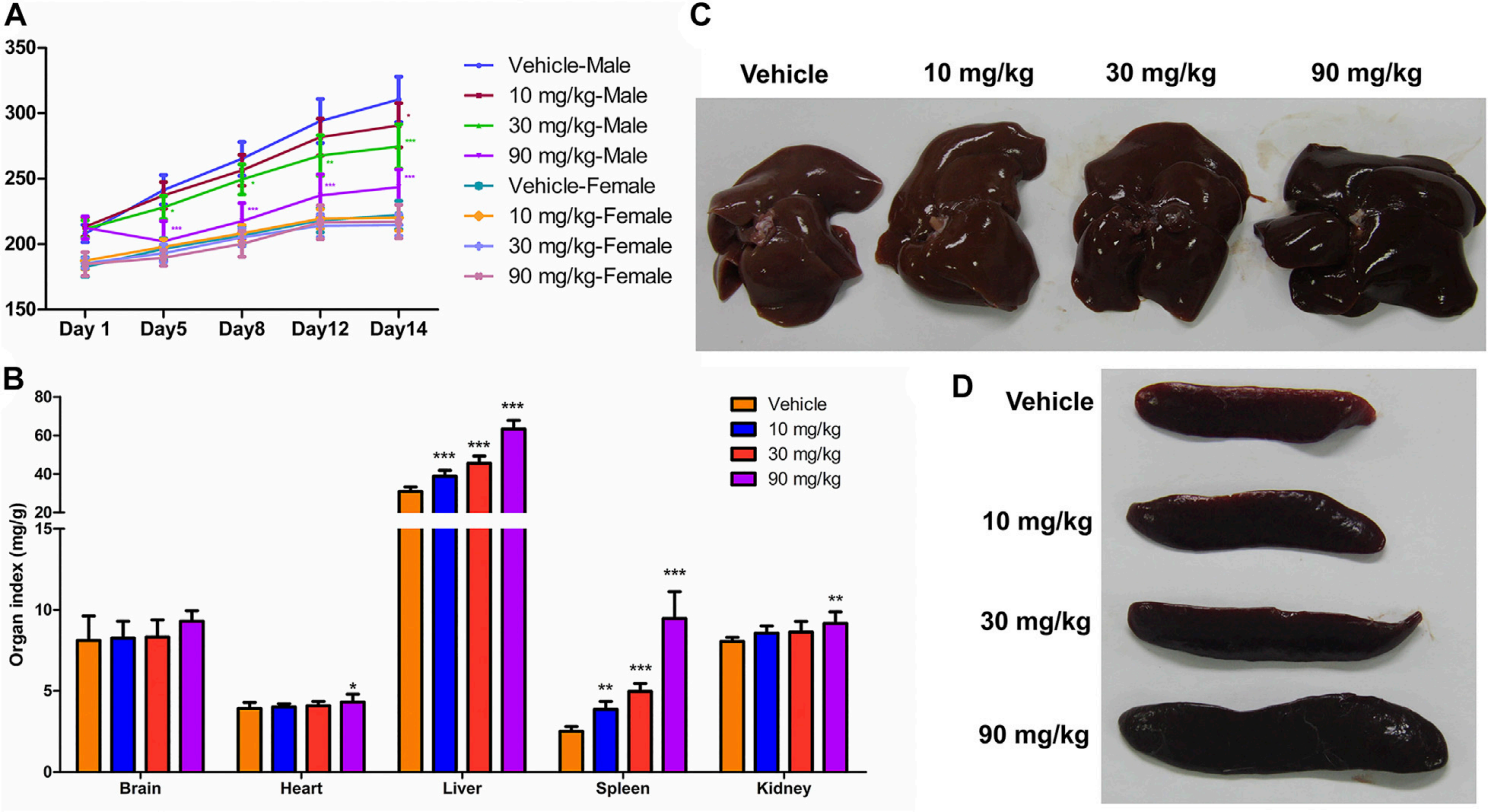
General physiological findings and organ coefficients after IONPs administration: **(A)** Changes in the body weight of male and female rats treated with iron oxide nanoparticles (IONPs) for 14 days. Results are expressed as means ± standard deviation (*n* = 11). **(B)** Organ coefficients for the brain, heart, liver, spleen, and kidneys in rats administered indicated doses of IONPs relative to those administered the vehicle. Results are expressed as means ± standard deviation (*n* = 10). **(C,D)** Representative images of the liver and spleen following the administration of vehicle or IONPs for 14 days. *p < 0.05, **p < 0.01, ***p < 0.001 versus vehicle.

The body weights continually increased during the experimental period, and the body weight gain was lower in the IONPs-administered male rats than in the vehicle-administered male rats (Figure 1A). At the end of the experimental period, the average body weights of the male rats in the 10, 30, and 90 mg/kg IONPs groups were significantly decreased than in the vehicle group with 0.94-, 0.88-, and 0.78-fold of the average body weight, respectively, especially in later two IONPs groups (*p* < 0.001). Significantly decreased food intake was noted in the 90 mg/kg IONPs group, which was correlated with lower body weight gain (data not shown).

### 3.3 Organ coefficients

The organ coefficients, which were determined based on the terminal organ and body weights following an overnight fasting period prior to necropsy, were used as a highly sensitive measurement of toxicity reflecting significant changes even before the observation of histological changes. The organ coefficients of the heart and kidneys were higher in the 90 mg/kg IONPs group than in the vehicle group (*p* < 0.05), and the organ coefficients of the liver and spleen were significantly higher in all IONPs groups than in the vehicle group (*p* < 0.01) in a dose-dependent manner (Figures 1B–D).

### 3.4 Clinical chemistry

The clinical chemistry data of the male and female rats are summarized in Table 1. Briefly, the levels of total bile acids, cholesterol, triglycerides, and lipase were significantly increased in both the male and female rats administered 90 mg/kg IONPs compared to the vehicle group, which suggested the effect of IONPs on lipid metabolism. All other alterations in clinical chemistry parameters, including those exhibiting statistically significant differences, were not considered to be IONPs toxicity because the changes were negligible in magnitude, lacked dose dependency, were within normal biological variation, or were without toxicological significance.

**TABLE 1:** Clinical chemistry parameters of rats treated with/without IONPs for 14 days.

Parameters	Vehicle		10 mg/kg		30 mg/kg		90 mg/kg	
	Male	Female	Male	Female	Male	Female	Male	Female
Liver profile								
Total protein (g/L)	50.2 ± 0.6	54.9 ± 1.9	50.1 ± 2.6	53.1 ± 6.3	49.0 ± 4.5	51.1 ± 2.9	58.1 ± 1.6**	56.2 ± 2.9
Albumin (g/L)	28.1 ± 0.4	31.2 ± 0.7	27.0 ± 0.6	28.3 ± 3.1	26.2 ± 2.3	26.5 ± 1.0**	27.4 ± 1.3	26.8 ± 1.0**
Globulin (g/L)	22.1 ± 0.6	23.7 ± 1.2	23.1 ± 2.0	24.8 ± 4.2	22.7 ± 2.3	24.6 ± 2.5	30.6 ± 1.1***	29.3 ± 2.1*
ALP (U/L)	181 ± 31	94 ± 31	144 ± 27	96 ± 16	126 ± 31*	87 ± 53	201 ± 25	191 ± 43**
ALT (U/L)	36 ± 5	25 ± 4	33 ± 8	23 ± 2	30 ± 9	23 ± 3	52 ± 35	25 ± 5
AST (U/L)	166 ± 63	129 ± 30	106 ± 13	127 ± 20	93 ± 19	111 ± 21	172 ± 94	129 ± 30
Total bilirubin (μmol/L)	0.62 ± 0.14	0.47 ± 0.18	0.95 ± 0.30	0.52 ± 0.16	0.83 ± 0.25	0.63 ± 0.19	1.64 ± 0.19	1.46 ± 0.74**
Total bile acid (μmol/L)	5.6 ± 1.4	55.3 ± 6.5	6.2 ± 2.5	68.1 ± 9.7	6.0 ± 4.0	70.8 ± 7.8	24.3 ± 9.3***	79.5 ± 16.0*
Renal profile								
Creatinine (μmol/L)	37.8 ± 2.4	47.5 ± 2.2	39.0 ± 2.7	41.4 ± 3.3	38.8 ± 4.2	41.8 ± 4.8	45.9 ± 2.2**	37.0 ± 4.2**
Blood urea (mmol/L)	5.12 ± 0.73	4.87 ± 0.69	5.04 ± 0.51	4.16 ± 0.41	5.15 ± 1.08	3.95 ± 0.48	6.05 ± 0.74	4.91 ± 0.96
Cardiac profile								
Creatine kinase (U/L)	820 ± 393	762 ± 273	474 ± 61*	810 ± 401	345 ± 127**	416 ± 118	334 ± 56**	404 ± 116
LDH (U/L)	1,600 ± 507	1,557 ± 506	1,017 ± 235	1,613 ± 382	649 ± 296	1,144 ± 248	582 ± 104	951 ± 232
Lipid profile								
Cholesterol (mmol/L)	1.20 ± 0.29	1.54 ± 0.21	1.64 ± 0.24	1.78 ± 0.32	2.22 ± 0.34***	2.11 ± 0.44	2.68 ± 0.26***	2.99 ± 0.52***
Triglyceride (mmol/L)	0.39 ± 0.11	0.27 ± 0.07	0.82 ± 0.47	0.29 ± 0.03	0.99 ± 0.08*	0.57 ± 0.19**	0.75 ± 0.22	0.63 ± 0.16**
Lipase (U/L)	26 ± 1	28 ± 2	28 ± 1	28 ± 1	30 ± 1**	31 ± 2	38 ± 3***	38 ± 2***
Serum electrolytes								
Na+ (mmol/L)	144.1 ± 0.6	145.1 ± 2.0	143.8 ± 1.7	144.5 ± 2.6	143.3 ± 1.1	144.8 ± 0.5	142.3 ± 1.4	144.5 ± 2.3
K+ (mmol/L)	4.66 ± 0.27	4.54 ± 0.36	4.19 ± 0.32	4.74 ± 0.43	4.11 ± 0.36*	4.76 ± 0.65	3.97 ± 0.26**	4.49 ± 0.52
Cl- (mmol/L)	109.0 ± 2.6	102.6 ± 0.4	106.8 ± 2.4	102.2 ± 1.7	107.6 ± 2.8	101.4 ± 2.7	103.2 ± 2.0**	101.0 ± 1.2
Ca (mmol/L)	2.44 ± 0.07	2.50 ± 0.05	2.34 ± 0.06	2.45 ± 0.11	2.38 ± 0.07	2.42 ± 0.12	2.47 ± 0.06	2.41 ± 0.05
P (mmol/L)	2.96 ± 0.19	2.54 ± 0.25	2.67 ± 0.15*	2.41 ± 0.09	2.54 ± 0.13***	2.50 ± 0.18	2.65 ± 0.09**	2.36 ± 0.13
Mg (mmol/L)	0.76 ± 0.03	0.79 ± 0.02	0.74 ± 0.01	0.79 ± 0.02	0.72 ± 0.02	0.78 ± 0.03	0.73 ± 0.02	0.77 ± 0.02
Glucose profile								
Glucose (mmol/L)	6.65 ± 1.24	6.42 ± 0.71	5.50 ± 1.05	7.61 ± 1.43	6.73 ± 0.24	6.94 ± 0.66	6.68 ± 0.40	6.65 ± 0.52

Notes: Values are means ± standard deviation (*n* = 5).

* *p* < 0.05.

** p < 0.01.

*** *p* < 0.001 vs. vehicle.

Abbreviations: ALP, alkaline phosphatase; ALT, alanine aminotransferase; AST, aspartate aminotransferase; LDH, lactate dehydrogenase.

### 3.5 Hematology and coagulation

The total leucocyte number and the neutrophil percentage were significantly higher and the percentage of lymphocytes was significantly lower in the 90 mg/kg IONP group than in the vehicle group (Table 2). Aforementioned results suggested that the administration of 90 mg/kg IONPs induced a mild inflammatory response dominated by neutrophils. A significant decrease in mean corpuscular hemoglobin concentration was observed in the female rats in the 30 and 90 mg/kg IONPs groups, although the magnitude was small and within the range of the historical control data. The increase in fibrinogen noted in the 90 mg/kg IONPs group was not considered toxicologically meaningful because of the absence of differences in other coagulation parameters. The other changes noted in hematology and coagulation parameters were not considered to be related to IONPs administration because of the inconsistent direction of changes or the lack of any relationship to dose.

**TABLE 2:** Hematological parameters of rats treated with/without IONPs for 14 days.

Parameters	Vehicle		10 mg/kg		30 mg/kg		90 mg/kg	
	Male	Female	Male	Female	Male	Female	Male	Female
WBC (10^^9^/L)	4.42 ± 1.81	3.81 ± 1.16	8.14 ± 1.43*	3.91 ± 1.30	6.48 ± 1.91	6.43 ± 1.63	13.33 ± 2.21***	10.65 ± 3.05***
Ne (%)	13.4 ± 5.7	11.2 ± 4.8	15.3 ± 3.0	13.8 ± 5.5	16.6 ± 5.4	18.7 ± 5.3	30.5 ± 7.2***	24.2 ± 5.3**
Mo (%)	9.7 ± 3.6	8.1 ± 2.1	9.0 ± 1.4	9.9 ± 2.8	10.2 ± 2.5	10.1 ± 1.3	14.5 ± 4.0	10.5 ± 2.7
Ly (%)	76.0 ± 9.5	79.2 ± 7.0	75.1 ± 4.2	75 ± 7.4	72.4 ± 6.7	70.1 ± 5.2	54.1 ± 9.4**	64.1 ± 6.3**
Eo (%)	0.7 ± 0.5	1.1 ± 0.2	0.5 ± 0.3	1.0 ± 0.4	0.7 ± 0.4	0.9 ± 0.2	0.6 ± 0.3	0.9 ± 0.4
RBC (10^^12^/L)	6.56 ± 0.51	6.76 ± 0.35	6.67 ± 0.21	6.03 ± 0.52*	7.07 ± 0.45	6.23 ± 0.39	6.35 ± 0.38	6.47 ± 0.18
HGB (g/L)	134 ± 7	138 ± 7	137 ± 5	124 ± 6**	146 ± 4*	130 ± 4	131 ± 7	136 ± 2
HCT (%)	38.9 ± 1.8	39.5 ± 1.6	39.6 ± 0.8	36.3 ± 2.0*	41.9 ± 1.2**	38.3 ± 1.1	37.4 ± 1.5	40.0 ± 1.0
MCV (fL)	59.4 ± 2.4	58.4 ± 1.9	59.4 ± 0.8	60.3 ± 1.9	59.3 ± 2.7	61.5 ± 3.1	59.0 ± 1.7	61.8 ± 2.3
MCHC (g/dl)	34.4 ± 0.7	34.9 ± 0.6	34.6 ± 0.7	34.1 ± 0.4	34.9 ± 0.2	34.0 ± 0.7*	34.9 ± 1.0	33.9 ± 0.3*
PLT (10^^9^/L)	1,068 ± 88	1,142 ± 75	1,470 ± 178***	1,085 ± 577	1,245 ± 135	1,404 ± 158	1,037 ± 93	1,440 ± 112
MPV (fL)	7.5 ± 0.4	7.1 ± 0.2	7.1 ± 0.1	7.2 ± 0.4	7.2 ± 0.1	6.9 ± 0.2	7.6 ± 0.4	7.1 ± 0.1
Coagulation								
Fbg (g/L)	2.19 ± 0.11	1.79 ± 0.21	2.56 ± 0.09	2.26 ± 0.51	2.53 ± 0.12	2.30 ± 0.63	3.68 ± 0.54***	2.78 ± 0.31**
PT (s)	9.5 ± 0.3	7.4 ± 0.2	9.4 ± 0.9	7.2 ± 0.2	11.1 ± 1.6	7.1 ± 0.1*	9.1 ± 0.7	7.2 ± 0.1
APTT (s)	18.9 ± 1.1	12.7 ± 1.6	16.6 ± 3.7	13.8 ± 1.9	21.3 ± 2.8	13.3 ± 3.4	16.1 ± 3.0	12.5 ± 1.2
TT (s)	34.8 ± 1.7	30.3 ± 2.5	31.7 ± 5.3	29.5 ± 0.5	35.4 ± 4.9	30.1 ± 2.8	33.9 ± 1.8	30.1 ± 2.0

Notes: Values are means ± standard deviation (*n* = 5).

* *p* < 0.05.

** *p* < 0.01.

*** *p* < 0.001 vs. vehicle.

Abbreviations: WBC, white blood cell count; Ne, neutrophil; Mo, monocyte; Ly, lymphocyte; Eo, eosinophil RBC, red blood cell count; HGB, hemoglobin; HCT, hematocrit; MCV, mean corpuscular volume; MCHC, mean corpuscular hemoglobin concentration; PLT, platelet count; MPV, mean platelet volume; Fbg, fibrinogen; PT, prothrombin time; APTT, activated partial thromboplastin time; TT, thrombin time.

### 3.6 Histopathology

**FIGURE 2:**
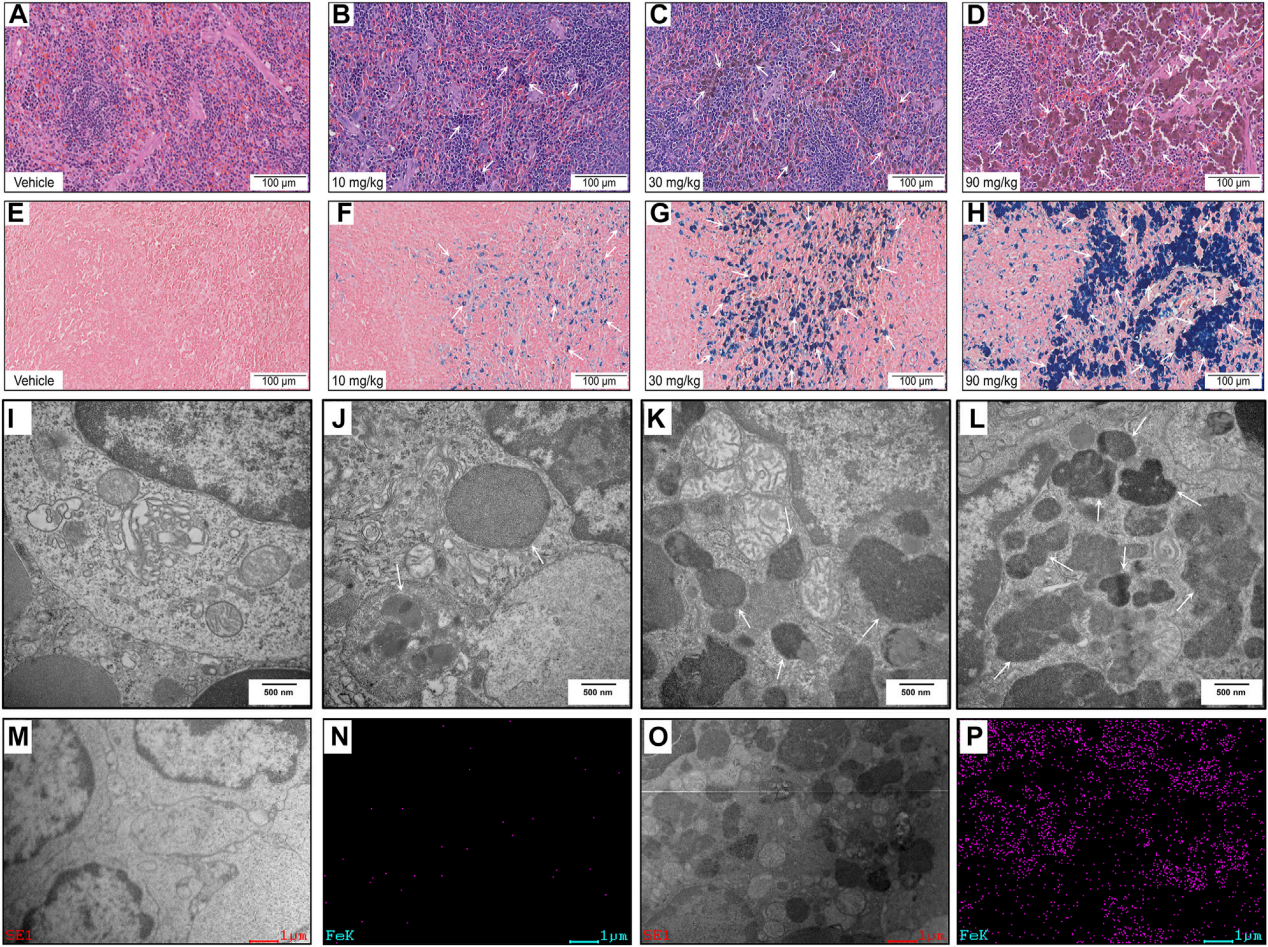
Histopathology and ultrastructural alterations in the spleen: (A–H) Representative images of specimens stained with hematoxylin and eosin **(A–D)** and Perls’ Prussian blue **(E–H)** after treatment with vehicle or IONPs for 14 days. **(I–L)** Representative transmission electron microscopy (TEM) images of spleen specimens following treatment with vehicle or IONPs for 14 days. **(M–P)** TEM-energy-dispersive spectrometry (EDS) analysis of the spleen after treatment with vehicle or 90 mg/kg IONPs for 14 days; **(M,O)** and **(N,P)** show the TEM and corresponding iron element distribution in the EDS images of the spleens after treatment with vehicle and IONPs, respectively. The EDS images show the element distribution of iron in the entire TEM image.

The histopathological findings are summarized in Supplementary Table S1. IONPs induced dose-dependent pigmentation in multiple organs, especially in the spleen (Figures 2A–D, arrows), and no apparent cellular structural changes were noted at any dose level. The pigmentation of macrophages in the spleen was confirmed as ferric iron using Perls’ Prussian blue staining (Figures 2E–H, arrows).

### 3.7 Subcellular iron oxide nanoparticles localization and ultrastructural alterations in the spleen

TEM was used to investigate the subcellular localization of the IONPs and the ultrastructural alterations in the spleen. Hypertrophied macrophages exhibited IONPs incorporated within the phagolysosomes. Dose-dependent accumulation of degradative autophagic vacuoles (AVDs) was noticeable in macrophages (Figures 2I–L, arrows). The corresponding EDS spectra of whole areas in the 90 mg/kg IONPs group indicated that the element iron was included in the AVDs with high electron density (Figures 2M–P). Iron was not detected in other areas without vesicles in the IONPs groups or the vehicle group. However, other ultrastructural features were normal, including mitochondria and normal nucleus with regular nuclear membranes and nucleopores.

### 3.8 Toxicokinetics and tissue distribution

**FIGURE 3:**
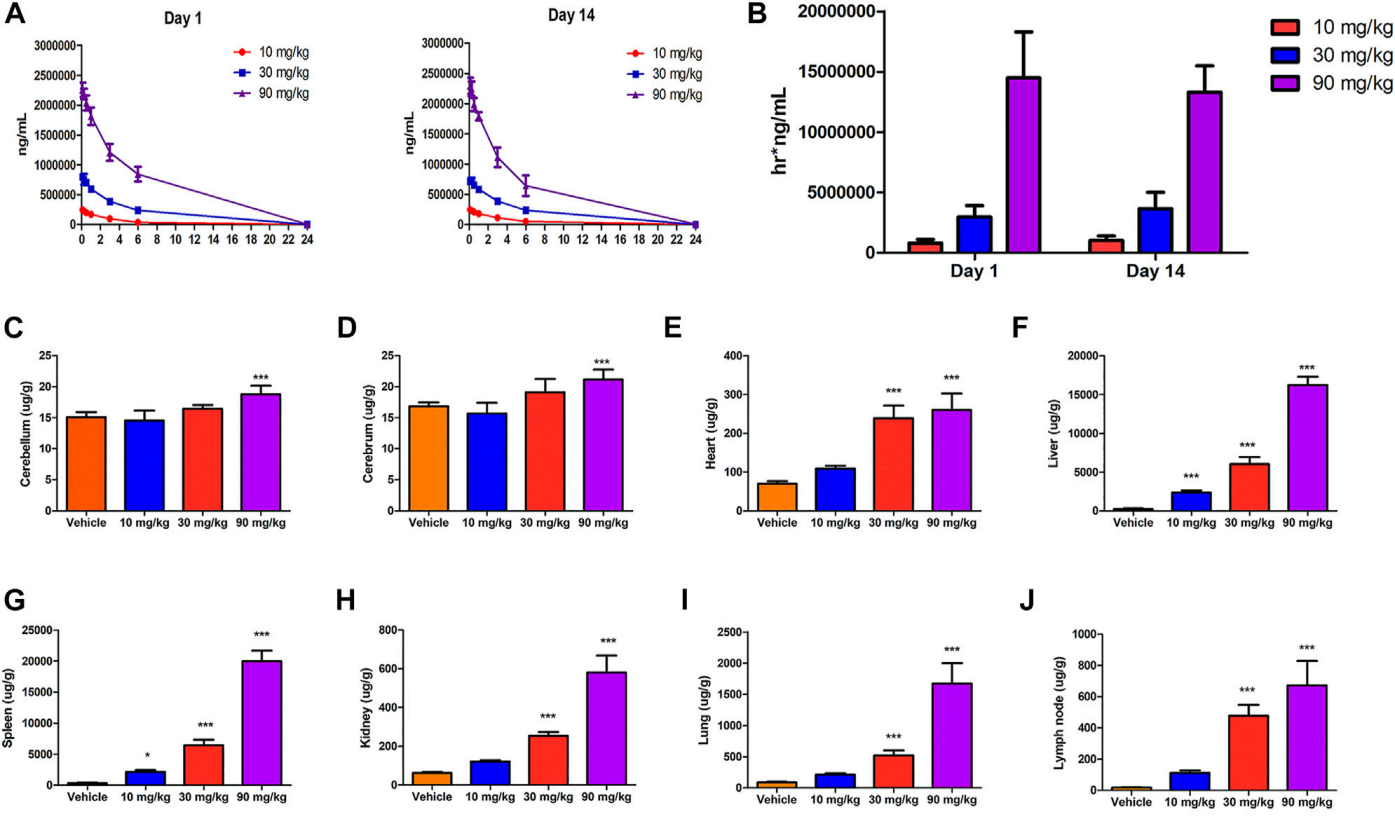
Toxicokinetics and tissue distribution after IONPs administration: **(A)** Mean serum concentration–time curves of IONPs after the first (day 1) and last doses (day 14). **(B)** The area under the concentration–time curve on days 1 and 14 of IONPs administration at different doses **(C–J)** Organ-specific distribution of IONPs after administration for 14 consecutive days. All results are expressed as means ± standard deviation (*n* = 6). *p < 0.05, **p < 0.01, ***p < 0.001 vs. vehicle.

Sex-related differences in toxicokinetics were not observed following systemic exposure to IONPs, and the male/female ratio for C_max_ and area under the curve (AUC) were less than two-fold. Therefore, the toxicokinetic parameters and the mean concentration–time curves shown in Figures 3A,B include the combined data of male and female animals. After intravenous administration of IONPs, T_max_ appeared at 5 min postdose, T_1/2_ was at approximately 2–4 h, and the mean residence time was within the range of 2–4 h. Systemic exposure expressed as C_max_ and AUC increased with increasing IONPs doses from 10 to 90 mg/kg close to or greater than the dose-proportion manner on days 1 and 14. IONPs accumulation after administration for 14 consecutive days was not observed at any dose. Tissue distribution analysis suggested that iron translocated into a range of tissues throughout the body, including the brain (cerebellum and cerebrum), heart, liver, spleen, kidneys, lungs, and mesenteric lymph nodes (Figures 3C–J). A dose-dependent increase in iron levels was observed in the spleen, liver, lungs, lymph nodes, kidneys, and heart. The accumulation pattern in organs was as follows: spleen > liver > lungs > mesenteric lymph nodes > kidneys > heart > brain.

### 3.9 Proteomics analysis of differentially expressed proteins in the spleen after iron oxide nanoparticles administration

#### 3.9.1 Protein identification

Traditional toxicological analyses indicated that spleen was the primarily affected organ, which was also involved in elimination after the intravenous administration of IONPs. However, no study has studied proteins with differential abundance in the spleen after IONPs exposure *in vivo*. Therefore, we used the TMT-based proteomics to investigate the changes in protein abundance in the spleen between the 90 mg/kg IONPs administration and vehicle groups.

**FIGURE 4:**
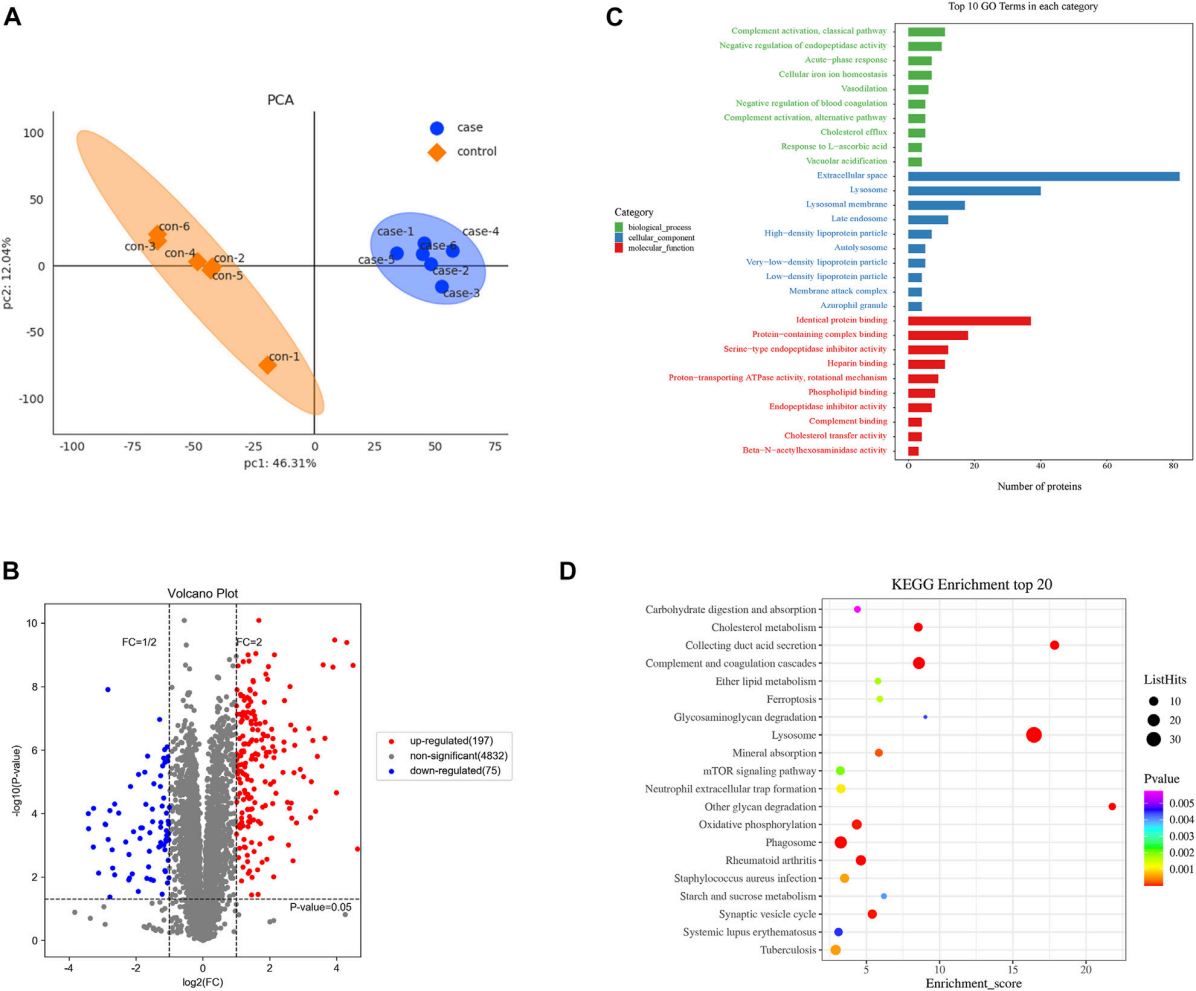
Proteomics analysis of differentially expressed proteins in the spleen after IONPs administration: **(A)** Principal Component Analysis of case (90 mg/kg IONPs) and control (vehicle) samples. **(B)** Volcano plots showing the number of differentially abundant proteins and the distribution of significance and fold change between the vehicle and 90 mg/kg IONPs groups. The gray dots are proteins with nonsignificant difference in expression, and the red and blue dots indicate significantly upregulated and downregulated proteins, respectively. **(C)** The Gene Ontology (GO) enrichment analysis graph showing top 10 terms enriched by *p* values in each of the ontological categories of biological process, cellular component, and molecular function in the 90 mg/kg IONPs group. **(D)** The Kyoto Encyclopedia of Genes and Genomes pathways enriched by DEPs identified between the 90 mg/kg IONPs and vehicle groups. The ListHits indicates the number of differentially abundant proteins enriched in specific pathways.

The proteomes of the different administration groups were compared using Principal Component Analysis (PCA). As shown in Figure 4A, the PCA scatter plot showed differences in the DEPs profile between the 90 mg/kg IONPs and vehicle groups.

A total of 5,269 proteins were identified, and 5,104 proteins were quantified in the vehicle and IONPs groups. Among these, 272 DEPs, including 197 upregulated and 75 downregulated proteins, were in the IONPs group based on the comparison with the vehicle group (Figure 4B).

#### 3.9.2 Gene Ontology functional enrichment analysis of differentially expressed proteins

To further analyze the functional characteristics, the DEPs were annotated and enriched using GO analysis and classified into three ontological categories of cellular component, biological process, and molecular function. The GO enrichment analysis graph in Figure 4C shows the top 10 items enriched by *p* values in each category.

Briefly, in the 90 mg/kg IONPs group, 268 DEPs were enriched in 1,571 terms in the biological process category, including 110 terms that were significantly enriched. The most abundant biological process terms were the complement activation classical pathway (GO: 0006958), negative regulation of endopeptidase activity (GO: 0010951), acute-phase response (GO: 0006953), cellular iron ion homeostasis (GO: 0006879), and vasodilation (GO: 0042311). In the cellular component category, 32 out of the 287 terms were significantly enriched and the five terms with the highest enrichment degree were extracellular space (GO: 0005615), lysosome (GO: 0005764), lysosomal membrane (GO: 0005765), late endosome (GO: 0005770), and high-density lipoprotein particle (GO: 0034364). Of a total of 442 terms enriched in the molecular function category, 37 were significantly enriched. The five most enriched terms were identical protein binding (GO: 0042802), protein-containing complex binding (GO: 0044877), serine-type endopeptidase inhibitor activity (GO: 0004867), heparin binding (GO: 0008201), and proton-transporting ATPase activity, rotational mechanism (GO: 0046961) (Figure 4C).

#### 3.1.3 Pathway enrichment analysis of differentially expressed proteins

We also analyzed the effect of IONPs 90 mg/kg administration on protein expression using KEGG pathway analysis. The analysis showed enrichment in 153 differentially abundant proteins in 226 pathways in the IONPs group, and 37 pathways were significantly enriched (*p* < 0.05). We noted significant enrichment in the expression of proteins involved in the lysosome, phagosome, rheumatoid arthritis, mTOR signaling pathway, oxidative phosphorylation, complement and coagulation cascades, collecting duct acid secretion, neutrophil extracellular trap formation and cholesterol metabolism were significantly enriched compared with the vehicle group, respectively. Overall, transport and catabolism, and the immune and excretory systems were significantly enriched by DEPs identified between the 90 mg/kg IONPs group and vehicle group (Figure 4D).

#### 3.9.4 Iron oxide nanoparticles administration leads to lysosomal activation and autophagy in splenic macrophages

**FIGURE 5:**
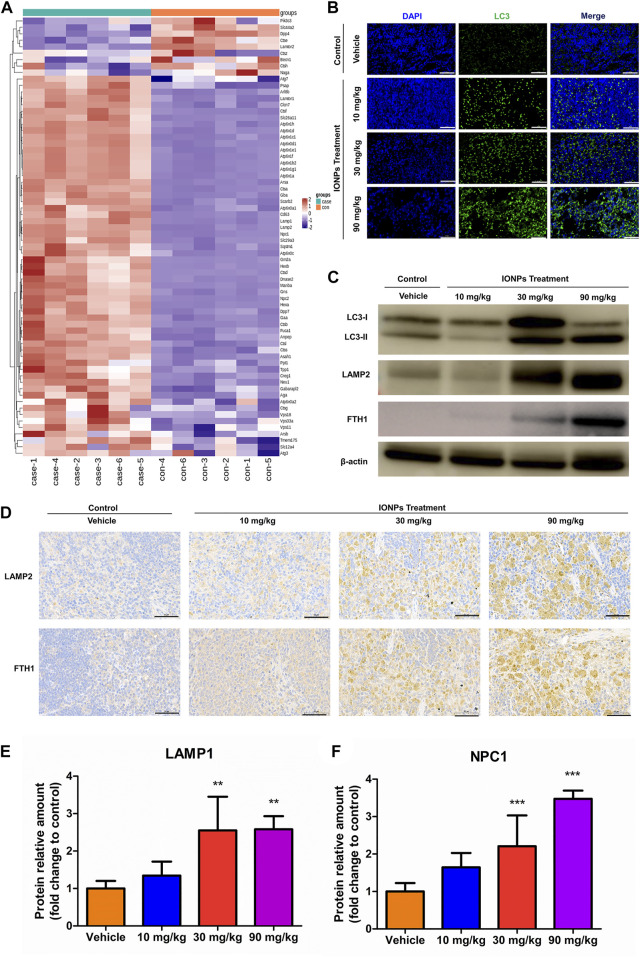
IONPs administration leads to lysosomal activation and autophagy in splenic macrophages: **(A)** Clustering heatmap of differentially expressed proteins associated with lysosomal activation **(B)** Immunofluorescence staining showing the expression of light chain 3 (LC3) in spleen specimens collected from rats treated with vehicle or different doses of IONPs. Nuclei are stained with DAPI. Scale bars: 50 μm. **(C)** The protein levels of LC3, FTH1, and LAMP2 were analyzed using western blotting, and *β*-actin was used as internal reference **(D)** Immunochemical staining showing the expression of ferritin heavy chain 1 (FTH1) and lysosome-associated membrane protein (LAMP) 2 in spleen specimens of rats treated with vehicle or different doses of IONPs. **(E)** and **(F)** The verification of LAMP1 and NPC1 abundance using parallel reaction monitoring. Data are shown as means ± standard deviation (*n* = 6). **p < 0.01, ***p < 0.001 vs. vehicle.

We analyzed proteomic data of 90 mg/kg spleen after IONPs administration using heatmaps and found significant differences in most proteins associated with lysosomal activation and autophagy (Figure 5A). The abundance of LAMP1, LAMP2, and NPC1 was significantly higher in the 30 and 90 mg/kg IONPs groups than in the vehicle group (Figures 5C–F). LAMP1 and LAMP2 play crucial roles in lysosomal enzyme targeting, autophagy, and lysosomal biogenesis (Fukuda, 1991; Eskelinen et al., 2003; Eskelinen et al., 2004; Huynh et al., 2007). NPC1 is a polytopic membrane protein located in the membranes of endosomes and lysosomes (Qian et al., 2020). TEM results showed dose-dependent increase of AVDs in splenic macrophages after IONPs administration. Therefore, these results indicated the lysosomal activation of splenic macrophages after IONPs administration. Meanwhile, the increase in LC3-II expression and the turnover of LC3-I to LC3-II indicated the activation of autophagy in spleen (Figures 5B,C). The activation of the lysosome-dependent autophagy can lead to IONPs degradation and to the liberate of free iron ions, which may trigger the excessive production of reactive oxygen species, cellular damage, and inflammatory response. FTH1 is the major protein responsible for the intracellular storage of iron in a soluble and nontoxic state. In the present study, the increase in FTH1 protein levels indicated that the degraded iron released from the IONPs was sequestered and stored in a nontoxic and bioavailable state (Figures 5C,D).

#### 3.9.5 The AKT/mTOR/TFEB signaling pathway facilitates in iron oxide nanoparticles-induced autophagy

**FIGURE 6:**
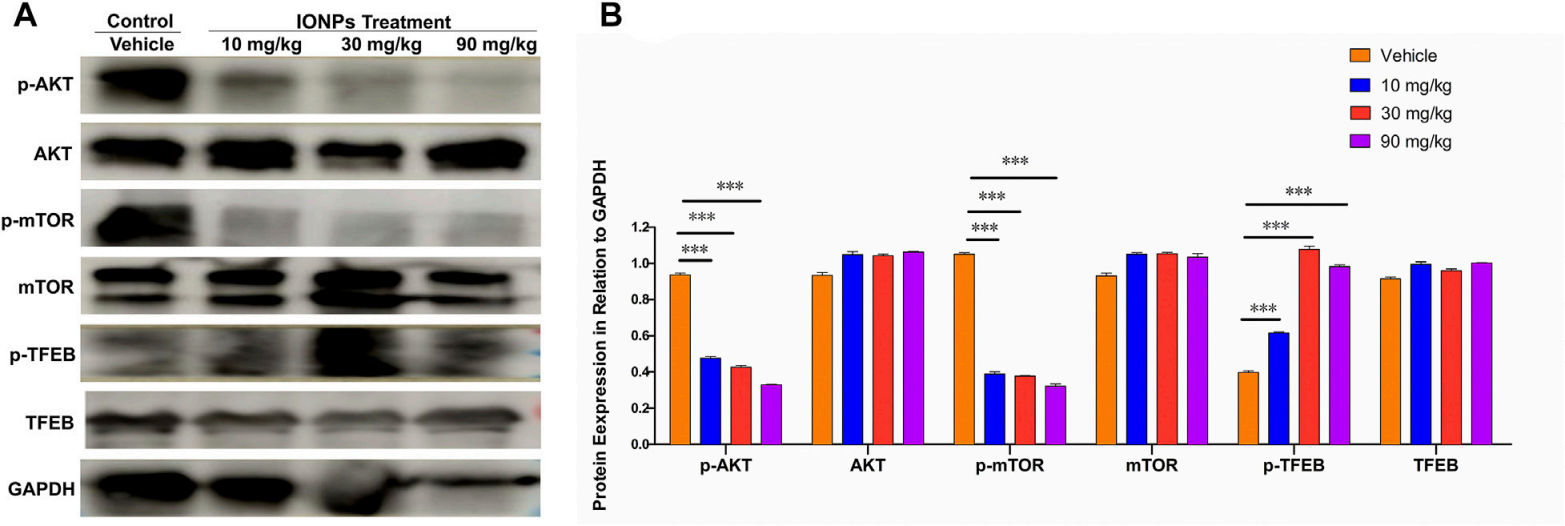
The AKT/mTOR/TFEB signaling pathway facilitates in IONPs-induced autophagy: **(A)** Representative western blotting image for p-ATK, AKT, p-mTOR, mTOR, p-TFEB, and TFEB in spleen samples of rats after IONPs administration. The protein expression levels of p-ATK, AKT, p-mTOR, mTOR, p-TFEB, and TFEB were semiquantitatively determined by measuring the integrated optical density of bands based on the western blot images. GAPDH was used as an internal reference. **(B)** Corresponding graph showing relative changes in protein expression levels. ***p < 0.001 vs. vehicle.

We also found that the IONPs-induced autophagy by inhibiting the AKT/mTOR signaling pathway in a dose-dependent fashion (Figures 6A,B). As an important transcription factor of autophagy downstream of the mTOR signaling pathway, TFEB in the spleen was significantly activated after IONPs administration. TFEB has been shown to be able to couple with autophagosomes to promote the maturation of LC3-driven autophagosomes and to regulate the fusion of autophagosomes with lysosomes. In the present study, we observed that the AKT/mTOR signaling pathway was inhibited and that the TFEB activity was significantly increased, which might be a primary mechanism by which IONPs induce autophagy in the spleen.

## 4 Discussion

IONPs have vast potential for utility in biomedical applications, especially magnetic resonance imaging (Liu et al., 2013; Dadfar et al., 2019). However, the toxicity profile of intravenously administered IONPs has not been fully elucidated. In the present study, we intravenously delivered IONPs in rats and evaluated toxicity and IONPs mechanisms using traditional toxicological assessment methods, such as physiological observations, clinical pathology, and histopathology, in combination with emerging proteomics analyses.

The established toxicological tests revealed that the administration of 90 mg/kg IONPs led to mild clinical signs and lower body weight gains in male rats. Serum biochemical analyses indicated that IONPs did not exert an obvious adverse effect on hepatic, renal, and cardiac functions. However, the increases in total bile acids, cholesterol, triglycerides, and lipase suggested a lipid metabolism disorder. Similarly, persistent iron-enriched diet fed to rats led to the impairment in the plasma lipid transport and hepatic sterol metabolism (Brunet et al., 1999). Dietary iron overload induces lipid peroxidation and increases in cholesterol and triglycerides, indicating the risk of hyperlipidemia. Meanwhile, the increase in total bile acids is related to cholesterol metabolism. Overall, these findings indicated that the altered lipid transport and cholesterol metabolism were related to the iron overload following IONPs administration. Hematological assessment revealed increases in leucocyte count and neutrophil percentage in the 90 mg/kg IONPs group, although there were no findings of evident inflammation during histopathological examination in entire organs. Neutrophils drive effecting mechanisms through phagocytosis, degranulation, and neutrophil extracellular traps (NET) formation (Burn et al., 2021). Studies investigating nanomaterial clearance by the immune system indicate that NETs act as physical barriers for nanoparticles (Bartneck et al., 2009). Consistently, Kuźmicka et al. demonstrated that excess iron increased the release of NETs and reactive oxygen species (Kuzmicka et al., 2022). Therefore, the observed increases in the leucocyte count and neutrophil percentage indicate a primary immune response and a potential mechanism of clearance of the IONPs from the systemic circulation after intravenous administration.

Toxicokinetic analysis showed that IONPs exhibited an appropriate elimination rate and a favorable dose–response relationship in the blood after intravenous administration. The tissue distribution results indicated that IONPs were delivered to a range of organ systems after entering the systemic circulation, in accordance with the results from numerous studies (Yu et al., 2018; Gaharwar et al., 2019; Bolandparvaz et al., 2020). In the present study, the highest IONPs deposition in the spleen and liver indicated that these two organs played the most important role in IONPs elimination from the systemic circulation. The increases in the size and organ coefficient of the spleen and liver were consistent with the iron levels obtained through the tissue distribution analysis. Both the spleen and the liver contain a large number of macrophages, including Kupffer cells in the liver, and are highly vascular, which are considered to exhibit prominent ability to actively capture the IONPs (Allard-Vannier et al., 2012; Barrefelt et al., 2013; Vercoza et al., 2019).

Histopathology findings were limited to pigmentation in multiple organs, which was confirmed as iron deposits using Perls’ Prussian blue staining, and cellular structural alterations were absent. These results demonstrated that IONPs administration had no adverse effect on microscopic morphology in any of the examined organs (Supplementary Table S1). Further ultrastructure examination of the spleen, which exhibited the highest iron accumulation, indicated that IONPs did not lead to subcellular structural changes, except for the dose-dependent increase in AVDs. TEM-EDS revealed the elemental enrichment of these AVDs as the subcellular distribution of the IONPs expressed as elemental iron. Our results are in accordance with previous *in vitro* studies in macrophages demonstrating the internalization of IONPs in a membrane-bound manner (Gu et al., 2011).

Proteomics is a powerful method to examine the mechanism of IONPs toxicity in studies evaluating their safety. The identification of the DEPs and the GO enrichment analysis revealed that the DEPs induced by the IONPs, compared with the vehicle group, were mainly involved in the cellular component category. Extracellular space, the GO term with the highest enrichment in the cellular component category, is potentially related to the gross pathological finding of increased spleen size (Mo, 2017). Complement activation, which was enriched in both the GO and KEGG pathway analyses, was not considered toxicologically meaningful because of the possible development of pseudoallergy by the rapid injection of iron-containing products (Rampton et al., 2014; Hempel et al., 2017). The significant enrichment of the term cholesterol metabolism was consistent with the changes in total bile acids, cholesterol, triglycerides, and lipase noted in serum biochemical analysis. NPC1, a multimembrane-spanning protein, is the central protein for lysosomal transfer of cholesterol (Meng et al., 2020). The trend in the protein expression was fully consistent with the results of the serum biochemistry tests. Therefore, the proteomics analyses used in the present study revealed the mechanisms of IONPs toxicity.

Numerous studies investigating nanoparticle uptake mechanisms have exhibited that endocytosis is the main route for the cellular entry of the nanoparticles and that the endocytic entry can be subdivided into the categories of phagocytosis, clathrin/caveolin-independent endocytosis, clathrin-mediated endocytosis, caveolin-mediated endocytosis, and micropinocytosis (Mailänder and Landfester. 2009; Nel et al., 2009; Francia et al., 2020; Yao et al., 2020). Phagosome was a highly enriched term in the KEGG pathway analysis of the proteomics results, demonstrating phagocytosis as the main mechanism of IONPs entry into splenic macrophages. Notably, the increase in AVDs and the abundance of lysosome-related terms noted in the GO and KEGG analyses indicate that lysosome-mediated degradation plays a crucial role in responses to IONPs administration. In fact, macrophages were shown to engulf and accumulate IONPs in lysosomes (Lunov et al., 2011a; Lunov et al., 2011b; Beddoes et al., 2015). The process for the intracellular biodegradation of IONPs can be quite long, and IONPs may retain in the body from several days to several weeks after intravenous injection (Lunov et al., 2010; Feng et al., 2018; Van de Walle et al., 2020). Therefore, it is plausible that IONPs substantially modulate lysosome-related signaling pathways. Many processes involved in cell death and pathological conditions have been shown to be associated with lysosomal dysfunction (Boya and Kroemer, 2008). Overall, our results revealed that the lysosome-related signaling pathways are the primary mechanism of IONPs toxicity in the spleen after intravenous administration.

The upregulation of both LAMP1 and LAMP2 confirmed in the present study indicates increases in lysosomal biogenesis and autophagic activity after IONPs administration. Notably, IONPs induce autophagy in numerous cultured cell types *in vitro* (Jin et al., 2019; Uzhytchak et al., 2020), which has not been thoroughly confirmed in animal models *in vivo*. Interestingly, the degree of autophagy, expressed as the turnover of the microtubule-associated protein LC3 (Mizushima et al., 2010), was significantly increased in a dose-related manner after IONPs administration in the present study. The AKT/mTOR signaling pathway inhibition has been reported to induce autophagy in human fetal neural stem cells fNSCs (Liang et al., 2016) and human lung cancer cells H460 cells (Fu et al., 2009) as well as the mouse mammary epithelial cells HC11 (Hou et al., 2020). mTOR is generally located on the lysosomal membrane, and TFEB in the cytoplasm is phosphorylated by mTOR under normal conditions, inducing mTOR activation (Settembre et al., 2012). Conversely, under pathological conditions, TFEB translocates from the cytoplasm to the nucleus, leading to the upregulation of autophagic proteins while mTOR is released from the lysosomal membrane, and the ensuing inhibition of mTOR activity can promote autophagy (Sardiello et al., 2009; Roczniak-Ferguson et al., 2012). In the present study, the AKT and mTOR activities in the spleen were significantly decreased after IONPs administration. The mTOR/TFEB signaling pathway, which is crucial for lysosomal biogenesis and autophagy activation, was significantly activated. Interestingly, the p-TFEB increased in the IONPs treated groups, but it was not higher in the 90 mg/kg group than in the 30 mg/kg group. This may because of the *in vivo* environment has been significantly altered by the administration of 90 mg/kg IONPs, which may reduce p-TFEB, consistent with in previous study (Ballabio, 2016). This is worth exploring more profound in future work. Autophagy is dependent on lysosomal degradation, which has been demonstrated to be an important regulator of various diseases and conditions (Mizushima and Levine, 2020; Jiang et al., 2021; Viret et al., 2021). Park et al. (2014) at Demonstrated that autophagy was an important protective mechanism of IONPs-induced apoptotic cell death and endoplasmic reticulum stress in RAW264.7 cells. Not surprisingly, rats treated with IONPs for 14 days did not exhibit severe inflammatory or toxic reactions based on the roles of autophagy in stress adaptation, immune, and inflammatory response (Cadwell, 2016). However, some studies showed that IONPs caused cell damage by inducing autophagy (Du et al., 2016; Zhang et al., 2020). Therefore, the role of autophagy in IONPs toxicity remains to be further elucidated.

Iron is an essential component of the human body, playing a crucial role in the activity of cytochromes, hemoproteins, and hemoglobin (Andrews, 2000). However, iron overload causes cell dysfunction and death, leading to tissue damage and organ dysfuntion (Trinder et al., 2002). FTH1 is a universally expressed and highly conserved protein that plays a major role in iron balance by sequestering and storing ions in a nontoxic and bioavailable manner (Munro et al., 1988; Edison et al., 2008). In the present study, FTH1 expression was significantly upregulated, suggesting the sequestration of iron released from the lysosomal IONPs degradation. Therefore, no obvious toxicity due to iron overload was noted after the intravenous IONPs administration in rats.

## 5 Conclusion

This is the first study utilizing a combination of traditional and emerging proteomics methods to assess IONPs toxicity following intravenous administration in rats and to elucidate underlying mechanisms. We showed that the lysosome is the master regulator of IONPs-mediated toxicity in spleen, the target organ of intravenously administered IONPs, and that the lysosome-related signaling pathways are the primary mechanism of toxicity in splenic macrophages. We also showed that the AKT/mTOR/TFEB signaling pathway facilitates in the IONPs-induced autophagy of splenic macrophages.

## Data Availability

The datasets presented in this study can be found in online repositories. The names of the repository/repositories and accession number(s) can be found below: ProteomeXchange Consortium (http://proteomecentral.proteo.mexchange.org) *via* the iProX partner repository with the dataset identifier PXD035970.
