# Supplementary material for: Proteomics unite traditional toxicological assessment methods to evaluate the toxicity of iron oxide nanoparticles
Source: Front Pharmacol. 2022 Sep 12;13:1011065. doi: 10.3389/fphar.2022.1011065 (PMC9512491; doi:10.3389/fphar.2022.1011065)
Supplement: Supplementary file 1 [file DataSheet1.docx]

Supplementary Material

# Supplementary Table 1

Summary of histopathological findings of rats treated with/without IONPs for 14 days

| Organ/Tissue | Histological  Finding | Grade | Vehicle | | 10 mg/kg | | 30 mg/kg | | 90 mg/kg | |
| --- | --- | --- | --- | --- | --- | --- | --- | --- | --- | --- |
|  |  |  | Male | Female | Male | Female | Male | Female | Male | Female |
| Mesenteric LN | Pigmentation，Macrophages | + | 0 | 0 | 5 | 5 | 0 | 0 | 0 | 0 |
|  |  | ++ | 0 | 0 | 0 | 0 | 5 | 5 | 4 | 1 |
|  |  | +++ | 0 | 0 | 0 | 0 | 0 | 0 | 1 | 4 |
|  |  | Incidence | 0/5 | 0/5 | 5/5 | 5/5 | 5/5 | 5/5 | 5/5 | 5/5 |
| Pituitary gland | Pigmentation | + | 0 | 0 | 5 | 5 | 5 | 5 | 0 | 0 |
|  |  | ++ | 0 | 0 | 0 | 0 | 0 | 0 | 5 | 4 |
|  |  | Incidence | 0/5 | 0/5 | 5/5 | 5/5 | 5/5 | 5/5 | 5/5 | 4/4* |
| Large intestinal | Pigmentation，Mucous layer | + | 0 | 0 | 0 | 0 | 5 | 5 | 5 | 5 |
|  |  | Incidence | 0/5 | 0/5 | 0/5 | 0/5 | 5/5 | 5/5 | 5/5 | 5/5 |
| Lung | Pigmentation，Macrophages | + | 0 | 0 | 5 | 5 | 0 | 0 | 0 | 0 |
|  |  | ++ | 0 | 0 | 0 | 0 | 5 | 5 | 0 | 0 |
|  |  | +++ | 0 | 0 | 0 | 0 | 0 | 0 | 5 | 5 |
|  |  | Incidence | 0/5 | 0/5 | 5/5 | 5/5 | 5/5 | 5/5 | 5/5 | 5/5 |
|  | Suppurative inflammation | +++ | 0 | 0 | 0 | 0 | 0 | 0 | 1 | 0 |
|  |  | Incidence | 0/5 | 0/5 | 0/5 | 0/5 | 0/5 | 0/5 | 1/5 | 0/5 |
| Liver | Pigmentation，kupffer cells | ++ | 0 | 0 | 5 | 5 | 0 | 0 | 0 | 0 |
|  |  | +++ | 0 | 0 | 0 | 0 | 5 | 5 | 0 | 0 |
|  |  | ++++ | 0 | 0 | 0 | 0 | 0 | 0 | 5 | 5 |
|  |  | Incidence | 0/5 | 0/5 | 5/5 | 5/5 | 5/5 | 5/5 | 5/5 | 5/5 |
|  | Pigmentation，Hepatocyte | ++ | 0 | 0 | 5 | 5 | 0 | 0 | 0 | 0 |
|  |  | +++ | 0 | 0 | 0 | 0 | 5 | 5 | 0 | 0 |
|  |  | ++++ | 0 | 0 | 0 | 0 | 0 | 0 | 5 | 5 |
|  |  | Incidence | 0/5 | 0/5 | 5/5 | 5/5 | 5/5 | 5/5 | 5/5 | 5/5 |
| Epididymis | Pigmentation，Stromatic | + | 0 | NA | 5 | NA | 5 | NA | 5 | NA |
|  |  | Incidence | 0/5 | NA | 5/5 | NA | 5/5 | NA | 5/5 | NA |
| Testis | Pigmentation，Stromatic | + | 0 | NA | 5 | NA | 5 | NA | 5 | NA |
|  |  | Incidence | 0/5 | NA | 5/5 | NA | 5/5 | NA | 5/5 | NA |
| Administration site  （tail veins） | Pigmentation，Hypodermic | + | 0 | 0 | 5 | 5 | 0 | 0 | 0 | 0 |
|  |  | ++ | 0 | 0 | 0 | 0 | 5 | 5 | 4 | 5 |
|  |  | Incidence | 0/5 | 0/5 | 5/5 | 5/5 | 5/5 | 5/5 | 4/4* | 5/5 |
| Femur | Pigmentation，Myeloid  macrophages | ++ | 0 | 0 | 5 | 5 | 5 | 5 | 0 | 0 |
|  |  | +++ | 0 | 0 | 0 | 0 | 0 | 0 | 5 | 5 |
|  |  | Incidence | 0/5 | 0/5 | 5/5 | 5/5 | 5/5 | 5/5 | 5/5 | 5/5 |
| Parathyroid gland | pigmentation | + | 0 | 0 | 5 | 5 | 5 | 5 | 5 | 5 |
|  |  | Incidence | 0/5 | 0/5 | 5/5 | 5/5 | 5/5 | 5/5 | 5/5 | 5/5 |
| Thyroid gland | pigmentation | + | 0 | 0 | 5 | 5 | 5 | 5 | 5 | 5 |
|  |  | Incidence | 0/5 | 0/5 | 5/5 | 5/5 | 5/5 | 5/5 | 5/5 | 5/5 |
| Submandibular LN | Pigmentation，Macrophages | + | 0 | 0 | 3 | 5 | 0 | 0 | 0 | 0 |
|  |  | ++ | 0 | 0 | 0 | 0 | 5 | 5 | 5 | 5 |
|  |  | Incidence | 0/5 | 0/5 | 3/3* | 5/5 | 5/5 | 5/5 | 5/5 | 5/5 |
| Spleen | Pigmentation，Macrophages | ++ | 0 | 0 | 5 | 5 | 0 | 0 | 0 | 0 |
|  |  | +++ | 0 | 0 | 0 | 0 | 5 | 5 | 0 | 0 |
|  |  | ++++ | 0 | 0 | 0 | 0 | 0 | 0 | 5 | 5 |
|  |  | Incidence | 0/5 | 0/5 | 5/5 | 5/5 | 5/5 | 5/5 | 5/5 | 5/5 |
|  | Atrophy，Marginal zone | + | 0 | 0 | 0 | 0 | 5 | 5 | 0 | 0 |
|  |  | ++ | 0 | 0 | 0 | 0 | 0 | 0 | 5 | 5 |
|  |  | Incidence | 0/5 | 0/5 | 0/5 | 0/5 | 5/5 | 5/5 | 5/5 | 5/5 |
| Ovary | Pigmentation，Corpus luteum | ++ | NA | 0 | NA | 5 | NA | 5 | NA | 1 |
|  |  | +++ | NA | 0 | NA | 0 | NA | 0 | NA | 4 |
|  |  | Incidence | NA | 0/5 | NA | 5/5 | NA | 5/5 | NA | 5/5 |
| Kidney | Pigmentation，Glomerulus | ++ | 0 | 0 | 5 | 5 | 0 | 0 | 0 | 0 |
|  |  | +++ | 0 | 0 | 0 | 0 | 5 | 5 | 0 | 0 |
|  |  | ++++ | 0 | 0 | 0 | 0 | 0 | 0 | 5 | 5 |
|  |  | Incidence | 0/5 | 0/5 | 5/5 | 5/5 | 5/5 | 5/5 | 5/5 | 5/5 |
| Adrenal | pigmentation | ++ | 0 | 0 | 5 | 5 | 5 | 5 | 0 | 0 |
|  |  | ++++ | 0 | 0 | 0 | 0 | 0 | 0 | 5 | 5 |
|  |  | Incidence | 0/5 | 0/5 | 5/5 | 5/5 | 5/5 | 5/5 | 5/5 | 5/5 |
| Stomach | Pigmentation，Mucous layer | + | 0 | 0 | 5 | 5 | 5 | 5 | 5 | 5 |
|  |  | Incidence | 0/5 | 0/5 | 5/5 | 5/5 | 5/5 | 5/5 | 5/5 | 5/5 |
| Small intestine | Pigmentation，Mucous layer | ++ | 0 | 0 | 5 | 5 | 5 | 5 | 5 | 5 |
|  |  | Incidence | 0/5 | 0/5 | 5/5 | 5/5 | 5/5 | 5/5 | 5/5 | 5/5 |
| Duodenum | Pigmentation，Mucous layer | ++ | 0 | 0 | 5 | 5 | 5 | 5 | 5 | 5 |
|  |  | Incidence | 0/5 | 0/5 | 5/5 | 5/5 | 5/5 | 5/5 | 5/5 | 5/5 |
| Sternum BM | Pigmentation，Macrophages | ++ | 0 | 0 | 0 | 0 | 5 | 5 | 0 | 0 |
|  |  | +++ | 0 | 0 | 0 | 0 | 0 | 0 | 5 | 5 |
|  |  | Incidence | 0/5 | 0/5 | 0/5 | 0/5 | 5/5 | 5/5 | 5/5 | 5/5 |
| vagina | Pigmentation，Stromatic | + | NA | 0 | NA | 5 | NA | 5 | NA | 5 |
|  |  | Incidence | NA | 0/5 | NA | 5/5 | NA | 5/5 | NA | 5/5 |
| Aorta | Pigmentation，Endotheliocyte | + | 0 | 0 | 5 | 5 | 5 | 5 | 5 | 5 |
|  |  | Incidence | 0/5 | 0/5 | 5/5 | 5/5 | 5/5 | 5/5 | 5/5 | 5/5 |
| Uterus | Pigmentation，Stromatic | + | NA | 0 | NA | 5 | NA | 5 | NA | 5 |
|  |  | Incidence | NA | 0/5 | NA | 5/5 | NA | 5/5 | NA | 5/5 |
| heart | Pigmentation，Stromatic | + | 0 | 0 | 0 | 0 | 0 | 0 | 2 | 0 |
|  |  | Incidence | 0/5 | 0/5 | 0/5 | 0/5 | 0/5 | 0/5 | 2/5 | 0/5 |

**Notes:** There were 5 rats/sex/dose examined. “*” indicate that individual animals missed the organ. Severity graded as grade + (minimal). grade ++ (mild), grade +++ (moderate), grade ++++ (marked)

**Abbreviations:** NA, not applicable; LN, lymph node; BM, bone marrow.

# Supplementary Figure 1


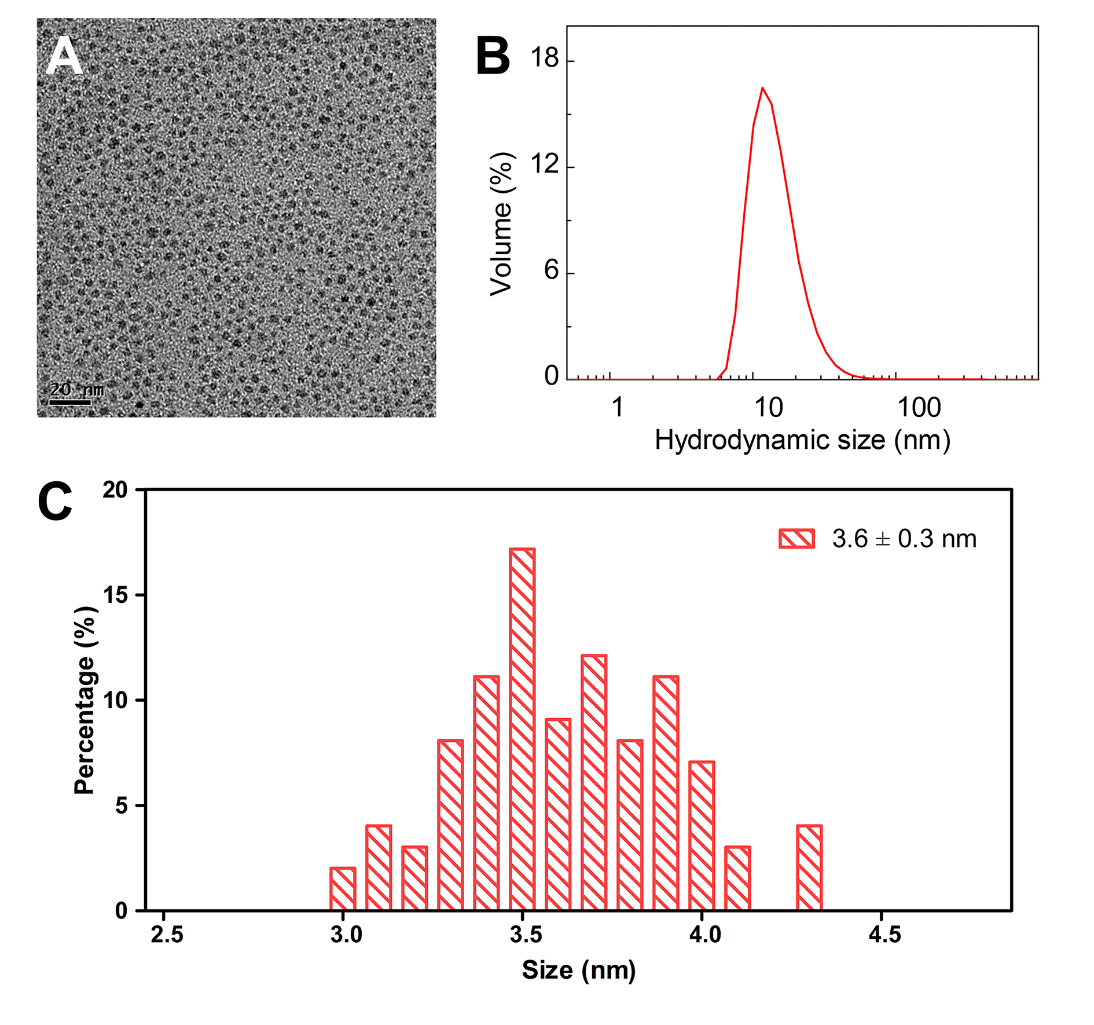


**Supplementary Figure 1.** Characterization of the injected IONPs: (A) The TEM images of the injected IONPs. (B) Hydrodynamic size distribution of the injected IONPs. (C) TEM size distribution of the injected IONPs.
